# Prognostic impact of shock at ICU admission in acute respiratory failure

**DOI:** 10.1371/journal.pone.0353556

**Published:** 2026-07-17

**Authors:** Tae Wan Kim, Jae Young Choi, Joonho Lee, Jin Song Park, Chi Ryang Chung, Jeong Hoon Yang, Gee Young Suh, Ryoung-Eun Ko

**Affiliations:** 1 Division of Pulmonary and Critical Care Medicine, Department of Internal Medicine, Chung-Ang University Hospital, Chung-Ang University College of Medicine, Seoul, Republic of Korea; 2 Department of Critical Care Medicine, Samsung Medical Center, Sungkyunkwan University School of Medicine, Seoul, Republic of Korea; AIIMS: All India Institute of Medical Sciences, INDIA

## Abstract

**Background:**

Acute respiratory failure (ARF) is a leading cause of intensive care unit (ICU) admission and is frequently complicated by shock. However, the prognostic significance of shock at ICU admission across the heterogeneous ARF population remains incompletely defined. We aimed to evaluate the association between shock at ICU admission and mortality in patients with ARF.

**Methods:**

This retrospective cohort study included 3,497 adult patients with ARF admitted to a tertiary medical center ICU in South Korea between January 2019 and December 2023. Shock was defined as the need for vasopressor or inotropic support at ICU admission. The primary outcome was hospital mortality, and the secondary outcome was ICU mortality. Multivariable Cox proportional hazards models were used to estimate adjusted hazard ratios (aHRs) with 95% confidence intervals (CIs).

**Results:**

Among 3,497 patients, 652 (18.6%) had shock at ICU admission. Patients with shock had significantly higher ICU mortality (46.4% vs. 17.1%) and hospital mortality (56.4% vs. 26.2%) compared with those without shock. After adjustment for potential confounders, shock remained independently associated with increased ICU mortality (aHR 1.84; 95% CI 1.56–2.17) and hospital mortality (aHR 1.58; 95% CI 1.37–1.82). This association was consistent across subgroups stratified by illness severity, respiratory support modality, and neutropenia status.

**Conclusion:**

Shock at ICU admission was independently associated with increased mortality in patients with ARF, with consistent findings across clinically relevant subgroups. The presence of shock at ICU admission may serve as a readily identifiable marker for risk stratification in this population.

## Introduction

Acute respiratory failure (ARF) is the most common indication for intensive care unit (ICU) admission and is associated with substantial mortality, with in-hospital mortality rates of approximately 33–37% among patients requiring mechanical ventilation [1–3]. Despite substantial advances in ventilatory strategies and supportive care, outcomes of ARF continue to be strongly influenced by the development of multiorgan dysfunction [2–4]. The clinical course of ARF is highly heterogeneous, reflecting differences in underlying etiology, baseline comorbidities, and the severity of acute physiological derangements at ICU admission [5,6].

In critically ill patients, ARF is frequently complicated by shock, highlighting the prognostic importance of other organ dysfunction beyond respiratory failure severity [7]. In patients with sepsis or severe pneumonia, shock is associated with rapid progression of organ dysfunction, metabolic derangements, and increased mortality [8–10]. Importantly, patients with ARF admitted to the ICU represent a clinically diverse population, including those supported with invasive mechanical ventilation as well as those managed with high-flow nasal cannula or noninvasive ventilation [11]. In this heterogeneous setting, the prognostic significance of shock remains incompletely understood. Moreover, previous studies have often focused on selected populations, limiting their generalizability to the broader spectrum of acute respiratory failure encountered in ICU patients [8,9].

Early risk stratification at ICU admission is critical for patients with ARF, as initial hemodynamic instability may signal systemic hypoperfusion and evolving extra-pulmonary organ dysfunction. However, the prognostic implications of shock present at ICU admission have not been systematically evaluated in broad ARF populations encompassing diverse etiologies and respiratory support modalities. Therefore, we aimed to investigate the association between shock at ICU admission and mortality in patients with ARF, and to assess the consistency of this association across clinically relevant subgroups.

## Materials and methods

### Study design and population

This retrospective cohort study included adult patients (≥ 18 years) with ARF who were admitted to a medical or cardiac ICU at a tertiary referral center in South Korea between January 2019 and December 2023. This study included all consecutive adult patients admitted to the ICU who required respiratory support meeting specific criteria. Patients were eligible if they received invasive mechanical ventilation (IMV) through either an endotracheal tube or a tracheostomy for more than 12 h. Additionally, patients who required advanced respiratory support, including high-flow nasal cannula (HFNC) or noninvasive ventilation (NIV) using bilevel positive airway pressure or continuous positive airway pressure with an oronasal or facial mask, were included if they demonstrated ARF, as characterized by either a partial pressure of arterial oxygen (PaO_2_)/ fraction of inspired oxygen (FiO_2_) ratio below 300 or a peripheral capillary oxygen saturation (SpO_2_)/FiO_2_ ratio below 315 for more than 1 hour [12,13]. As eligible patients could initiate respiratory support within 3 days of ICU admission rather than necessarily at ICU admission (day 0), SpO_2_/FiO_2_ values on day 0 were not available for all patients. Patients were excluded if they had a documented do-not-resuscitate order before or during ICU care or if they were under 18 years of age. A total of 3,497 patients with ARF were included in the final analysis.

Shock was defined as the need for vasopressor or inotropic support to maintain adequate tissue perfusion at ICU admission. Specifically, patients were classified as having shock if they required continuous infusion of norepinephrine, epinephrine, vasopressin, dopamine, dobutamine, or phenylephrine on ICU day 0. Patients were categorized into two groups based on the presence (n = 652) or absence (n = 2,845) of shock at ICU admission.

The reporting of this study followed the Strengthening the Reporting of Observational Studies in Epidemiology (STROBE) statement for cohort studies [14], and the completed STROBE checklist is provided as S1 File.

### Data collection and covariates

Data were extracted from the institutional clinical data warehouse, DARWIN-C, including clinical and laboratory variables on 31, July 2024. All data were de-identified prior to access, and the authors had no access to information that could identify individual participants at any time during or after data collection. Baseline data encompassed age, sex, body mass index, comorbidities, and the cause of respiratory failure. Sequential Organ Failure Assessment (SOFA) scores were recorded on ICU admission. Laboratory data were collected on day 0. Comorbidities assessed included chronic obstructive pulmonary disease (COPD), heart failure, ischemic heart disease, hypertension, diabetes mellitus, chronic kidney disease, stroke, osteoporosis, and malignancies (hematologic and solid tumors). Hypoxemia severity was classified using the SpO_2_/FiO_2_ ratio with validated thresholds corresponding to the Berlin Definition severity categories: mild (SpO_2_/FiO_2_ > 235), moderate (>148–235), and severe (≤148) [13].

Data on early ICU management within the first three days included corticosteroid use, respiratory support modalities, the application of continuous renal replacement therapy (CRRT) and veno-venous extracorporeal membrane oxygenation (VV-ECMO), vasoactive agents, and daily fluid balance.

### Study outcomes

The primary outcome was hospital mortality. The secondary outcome was ICU mortality. Outcomes were followed from ICU admission until hospital death or discharge, or until June 30, 2024, whichever occurred first.

### Statistical analysis

Continuous variables were expressed as medians with interquartile ranges (IQRs) and compared using Mann–Whitney U test or Student’s t-test depending on normality test result. Categorical variables were presented as counts and percentages and compared using the chi-square test or Fisher’s exact test, as appropriate based on expected cell counts. Missing data were not imputed. All analyses were performed on a complete-case basis (listwise deletion); each Cox proportional hazards model and subgroup analysis included patients with complete data for the variables entered in that specific model.

Kaplan–Meier survival curves were generated to compare hospital survival rates between two patient groups, with the time-to-event defined as the interval from ICU admission to in-hospital death. The log-rank test was used to assess statistical significance.

Cox proportional hazards models were used to estimate hazard ratios (HRs) and 95% confidence intervals (CIs) for ICU and hospital mortality associated with shock at ICU admission. Multivariable models were adjusted for covariates selected based on clinical relevance and potential confounding factors, including age, etiology of acute respiratory failure, SOFA score at ICU admission, comorbidities (hypertension, diabetes mellitus, stroke, chronic kidney disease, heart failure, and chronic obstructive pulmonary disease), and malignancy status (hematologic and solid tumors). The proportional hazards assumption was assessed using Schoenfeld residuals and was not violated.

Subgroup analyses were conducted to assess the consistency of the association between shock and hospital mortality across strata of SOFA score, respiratory support modality, neutropenia, malignancy, and etiologies of ARF. In patients with shock, univariable and multivariable Cox proportional hazards model was used to identify risk factors for hospital mortality. Subgroup analyses were exploratory. For each subgrouping variable, effect modification was assessed by adding a multiplicative interaction term to the Cox model, and no adjustment for multiple comparisons was applied. To assess the potential influence of the COVID-19 pandemic, we performed a sensitivity analysis restricted to patients admitted outside the pandemic period, which was defined as February 2020 through May 2023.

All analyses were performed using R (version 4.4.2; R Foundation for Statistical Computing, Vienna, Austria), and a two-sided *P* value < 0.05 was considered statistically significant.

### Ethics statement

The study was conducted in accordance with the Declaration of Helsinki and approved by the Institutional Review Board of Samsung Medical Center (IRB No. 2022-12-016.). The requirement for informed consent was waived due to the retrospective nature of the study using anonymized data.

## Results

### Baseline characteristics

Among the 3,497 patients with ARF admitted to the ICU and included in this study, 652 (18.6%) had shock at the time of ICU admission. The median age was similar between the two groups (65.0 years [IQR, 57.0–74.0] in patients with shock versus 65.0 years [IQR, 55.0–75.0] in patients without shock; *P* = 0.787), and patients with shock were more likely to be male (66.3% versus 60.6%, *P* = 0.008). Patients with shock presented with more severe illness on admission, as indicated by higher SOFA scores (10.0 [IQR, 7.0–13.0] versus 6.0 [IQR, 4.0–9.0], *P* < 0.001).

The most common cause of ARF in patients with shock was sepsis-related (49.2%), whereas pulmonary causes (32.2%) and cardiogenic causes (24.0%) were more prevalent in patients without shock (*P* < 0.001). Comorbidities such as stroke (9.4% versus 13.1%, *P* = 0.011), chronic kidney disease (11.5% versus 14.6%, *P* = 0.045), and heart failure (11.2% versus 16.2%, *P* = 0.002) were more common in patients without shock. In contrast, hematologic malignancy (16.7% versus 10.0%, *P* < 0.001) and oncologic malignancy (66.1% versus 50.2%, *P* < 0.001) were more prevalent among patients with shock. The distribution of hypoxemia severity, classified using the SpO_2_/FiO_2_ ratio, is presented in Table 1. Among patients with shock, 328 (50.3%) had mild (SpO_2_/FiO_2_ > 235), 161 (24.7%) had moderate (>148–235), and 163 (25.0%) had severe hypoxemia (≤148). The corresponding proportions among patients without shock were 1,444 (50.8%), 707 (24.9%), and 471 (16.6%), respectively. The distribution of hypoxemia severity differed significantly between the two groups (P < 0.001), with a higher proportion of severe hypoxemia observed in patients with shock.

**Table 1:** Baseline characteristics of acute respiratory failure patients with and without shock at ICU admission.

Variable	With shock (n = 652)	Without shock (n = 2845)	*P*
Age, years	65.0 (57.0 − 74.0)	65.0 (55.0 − 75.0)	0.787
Sex, male	432 (66.3)	1724 (60.6)	0.008
Body mass index, kg/m^2^	22.9 (20.4 − 25.6)	22.8 (20.2 − 25.7)	0.913
Reason for Acute respiratory failure			<0.001
Pulmonary cause	123 (18.9)	916 (32.2)	
Sepsis-related	321 (49.2)	300 (10.5)	
Cardiogenic cause	110 (16.9)	683 (24.0)	
Acute on chronic respiratory failure	3 (0.5)	35 (1.2)	
Post-operative	14 (2.1)	180 (6.3)	
Other	35 (5.4)	406 (14.3)	
Unclassified	46 (7.1)	325 (11.4)	
SOFA score	10.0 (7.0 − 13.0)	6.0 (4.0 − 9.0)	<0.001
Comorbidity			
Hypertension	439 (67.3)	1935 (68.0)	0.772
Diabetes	585 (89.7)	2377 (83.6)	<0.001
Chronic kidney disease	75 (11.5)	416 (14.6)	0.045
Oncologic malignancy	431 (66.1)	1429 (50.2)	<0.001
Hematologic malignancy	109 (16.7)	285 (10.0)	<0.001
Heart failure	73 (11.2)	460 (16.2)	0.002
Stroke	61 (9.4)	373 (13.1)	0.011
COPD	45 (6.9)	232 (8.2)	0.323
Osteoporosis	42 (6.4)	154 (5.4)	0.349
Coronary artery disease	17 (2.6)	106 (3.7)	0.200
Hypoxemia severity*			<0.001
Mild	328 (50.3)	1444 (50.8)	
Moderate	161 (24.7)	707 (24.9)	
Severe	163 (25.0)	471 (16.6)	
Not available	0 (0)	223 (7.8)	
Laboratory test on day 0			
White blood cell, x 10^3^/μL	8.1 (1.0 − 15.6)	10.1 (6.4 − 14.4)	<0.001
Hemoglobin, g/dL	9.0 (7.7 − 10.9)	10.0 (8.4 − 11.9)	<0.001
Platelet count, x 10^3^/μL	75.0 (35.0 − 144.0)	160.0 (84.0 − 234.0)	<0.001
Absolute neutrophil count, x 10^3^/μL	6.6 (0.6 − 13.7)	8.2 (4.7 − 12.2)	<0.001
INR	1.6 (1.4 − 2.2)	1.2 (1.1 − 1.5)	<0.001
Albumin, g/dL	2.8 (2.4 − 3.1)	3.1 (2.7 − 3.5)	<0.001
Total bilirubin, mg/dL	1.1 (0.7 − 2.5)	0.8 (0.5 − 1.3)	<0.001
AST, U/L	75.0 (32.0 − 286.5)	34.0 (22.0 − 74.0)	<0.001
ALT, U/L	40.0 (19.0 − 138.0)	24.0 (14.0 − 51.0)	<0.001
Blood urea nitrogen, mg/dL	32.0 (20.9 − 44.6)	23.2 (15.0 − 37.5)	<0.001
Creatinine, mg/dL	1.4 (0.9 − 2.2)	0.9 (0.6 − 1.7)	<0.001
C-reactive protein, mg/dL	12.4 (4.9 − 23.4)	6.4 (1.9 − 14.1)	<0.001
Procalcitonin, ng/mL	7.5 (1.7 − 38.0)	0.6 (0.2 − 2.8)	<0.001
Lactic acid, mmol/L	4.9 (2.7 − 9.2)	1.9 (1.3 − 3.2)	<0.001

Values are presented as median (interquartile range) or number (%).

*SpO_2_/FiO_2_ (S/F) ratio: severe ≤148, moderate >148–235, mild >235, S/F ratio could not be calculated in 223 patients without shock, owing to missing data at ICU admission.

Day 0 laboratory tests were performed at ICU admission.

Abbreviations: ALT, alanine aminotransferase; AST, aspartate aminotransferase; COPD, chronic obstructive pulmonary disease; HR, hazard ratio; ICU, intensive care unit; INR, international normalized ratio; SOFA, Sequential Organ Failure Assessment.

Patients with shock also presented greater systemic inflammation and organ dysfunction, as measured by elevated levels of C-reactive protein (12.4 versus 6.4 mg/dL), procalcitonin (7.5 versus 0.6 ng/mL), international normalized ratio (1.6 versus 1.2), total bilirubin (1.1 versus 0.8 mg/dL), creatinine (1.4 versus 0.9 mg/dL), aspartate aminotransferase (75.0 versus 34.0 U/L), alanine aminotransferase (40.0 versus 24.0 U/L), and lactate (4.9 versus 1.9 mmol/L), as well as lower hemoglobin (9.0 versus 10.0 g/dL), platelet counts (75.0 versus 160.0 × 10^3^/μL), and albumin levels (2.8 versus 3.1 g/dL) (all *P* < 0.001) (Table 1).

### ICU management and organ support

ICU management and organ support during the first 3 days are summarized in Table 2. A significantly higher proportion of patients with shock than those without shock received corticosteroids (82.7% versus 52.3%, respectively; *P* < 0.001). Hydrocortisone was the most commonly used corticosteroid in patients with shock (59.4%), followed by dexamethasone (17.3%) and methylprednisolone (6.0%). There was no significant difference in initial respiratory support modality between the two groups (*P* = 0.084); invasive mechanical ventilation was the most common modality in both groups (63.9% in patients with shock versus 59.1% in patients without shock).

**Table 2:** Early ICU management and organ support within the first 3 days according to shock status.

Variable	With shock (n = 652)	Without shock (n = 2845)	*P*
Corticosteroid use	539 (82.7)	1488 (52.3)	<0.001
Hydrocortisone	387 (59.4)	578 (20.3)	
Dexamethasone	113 (17.3)	690 (24.3)	
Methylprednisolone	39 (6.0)	220 (7.7)	
Initial respiratory support modality			0.084
Invasive mechanical ventilation	392 (63.9)	1529 (59.1)	
High-flow nasal cannula	213 (34.7)	1020 (39.4)	
Non-invasive ventilation	8 (1.3)	40 (1.5)	
CRRT	148 (22.7)	235 (8.3)	<0.001
Veno-venous ECMO	41 (6.3)	141 (5.0)	0.199
Prone positioning	45 (6.9)	136 (4.8)	0.035
Vasoactive or inotropic agents on day 0			
Norepinephrine	624 (95.7)	–	
Vasopressin	603 (92.5)	–	
Epinephrine	185 (28.4)	–	
Dobutamine	101 (15.5)	–	
Dopamine	93 (14.3)	–	
Phenylephrine	35 (5.4)	–	
Fluid intake on day 0, ml	3831 (2695 − 5227)	2404 (1744 − 3244)	<0.001
Fluid output on day 0, ml	2255 (1250 − 3453)	2260 (1480 − 3310)	0.373
Net fluid balance on day 0, ml	1429 (140 − 3038)	181 (−650 − 927)	<0.001
Fluid intake on day 1, ml	2989 (2217 − 4212)	2389 (1795 − 3128)	<0.001
Fluid output on day 1, ml	2492 (1380 − 3625)	2450 (1574 − 3435)	0.853
Net fluid balance on day 1, ml	467 (−360 − 1469)	−38 (−764 − 677)	<0.001
Fluid intake on day 2, ml	2747 (2097 − 3575)	2412 (1781 − 3214)	<0.001
Fluid output on day 2, ml	2795 (1725 − 3833)	2550 (1699 − 3515)	0.018
Net fluid balance on day 2, ml	29 (−763 − 900)	−115 (−815 − 548)	0.005

Values are presented as number (%) or median (interquartile range).

Day 0 refers to the day of ICU admission.

Fluid intake, output, and net fluid balance are calculated as the sum of all fluids administered or excreted in a 24-hour period (mL).

Abbreviations: CRRT, continuous renal replacement therapy; ECMO, extracorporeal membrane oxygenation; ICU, intensive care unit.

Use of CRRT was significantly more common in patients with shock (22.7% versus 8.3%, P < 0.001), and prone positioning was more frequently applied (6.9% versus 4.8%, *P* = 0.035). There was no significant difference in the use of VV-ECMO (6.3% versus 5.0%, *P* = 0.199). Among patients with shock, the most commonly used vasoactive agents on ICU day 0 were norepinephrine (95.7%) and vasopressin (92.5%), followed by epinephrine (28.4%), dobutamine (15.5%), dopamine (14.3%), and phenylephrine (5.4%). In addition, the net fluid balance on day 0 was significantly more positive in the shock group (1,429 mL [IQR, 140–3,038] versus 181 mL [IQR, −650–927], *P* < 0.001), with similar patterns observed on day 1 and 2.

### Clinical outcomes

**Fig 1:**
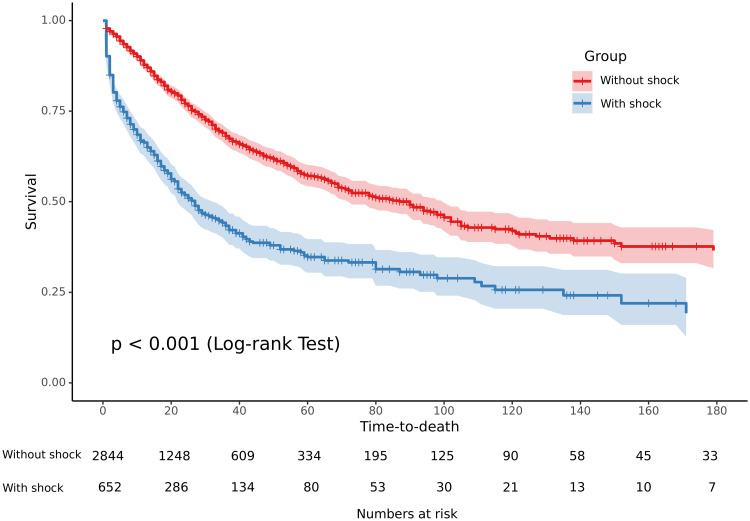
Kaplan–Meier survival curves for hospital mortality according to the presence of shock at ICU admission. Patients with shock (blue) demonstrated significantly lower survival probability compared with patients without shock (red) throughout the follow-up period (log-rank P < 0.001).

Shock at ICU admission was associated with significantly higher ICU and hospital mortality. ICU mortality was 46.4% (299/652) in patients with shock compared with 17.1% (434/2,845) in patients without shock (*P* < 0.001), and hospital mortality was 56.4% (368/652) versus 26.2% (744/2,845), respectively (*P* < 0.001) (Table 3). In univariable analysis, shock was associated with a 2.86-fold increased risk of ICU mortality (HR 2.86; 95% CI 2.47–3.31) and a 2.23-fold increased risk of hospital mortality (HR 2.23; 95% CI 1.97–2.53). After adjusting for potential confounders, shock remained independently associated with increased ICU mortality (adjusted HR 1.84; 95% CI 1.56–2.17; *P* < 0.001) and hospital mortality (adjusted HR 1.58; 95% CI 1.37–1.82; *P* < 0.001). Kaplan–Meier curves demonstrated significantly reduced hospital survival among patients with shock (log-rank *P* < 0.001) (Fig 1). In a sensitivity analysis restricted to the 963 patients admitted outside the COVID-19 pandemic period, the association between shock and hospital mortality remained significant (adjusted HR 1.58; 95% CI 1.23–2.05), consistent with the full cohort analysis (S2 Table). Complete univariable and multivariable analyses for all covariates are presented in S3 and S4 Table.

**Table 3:** Clinical outcomes of patients with and without shock.

Variable	With shock (n = 652)	Without shock (n = 2845)	Univariable analysis HR (95% CI)	Multivariable analysis aHR (95% CI)	*P*
ICU mortality	299 (46.4)	434 (17.1)	2.86 (2.47 − 3.31)	1.84 (1.56 − 2.17)	<0.001
Hospital mortality	368 (56.4)	744 (26.2)	2.23 (1.97 − 2.53)	1.58 (1.37 − 1.82)	<0.001

Values are presented as number (%) or median (interquartile range).

*P* values derived from a multivariable Cox proportional hazards model adjusted for age, reason for acute respiratory failure, Sequential Organ Failure Score, hematologic malignancy, oncologic malignancy, hypertension, diabetes, stroke, chronic kidney disease, heart failure, and chronic obstructive pulmonary disease.

Abbreviations: ICU, intensive care unit

### Subgroup analysis

**Fig 2:**
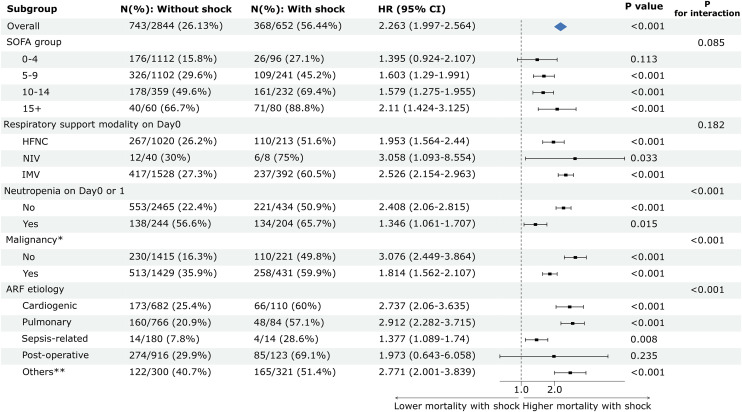
Forest plot showing the unadjusted hazard ratios for the association between shock and hospital mortality across clinical subgroups. The diamond represents the overall crude effect estimate. P values for interaction are shown to assess effect modification by each subgroup variable. *Malignancy includes both hematologic and solid malignancy. **Others include acute on chronic respiratory failure, neuromuscular disease, multiple trauma, and unclassified causes.

In subgroup analyses, unadjusted hazard ratios for hospital mortality according to shock status are presented in Fig 2, stratified by SOFA score category, respiratory support modality on day 0, neutropenia status, malignancy, and ARF etiologies. Overall, shock was significantly associated with increased hospital mortality (HR 2.263; 95% CI 1.997–2.564; P < 0.001). When stratified by illness severity, oxygen support device at ICU admission, and neutropenia status, shock remained consistently associated with higher hospital mortality across all examined subgroups. In a subgroup analysis stratified by malignancy status, shock remained significantly associated with increased hospital mortality in patients without malignancy (HR 3.076; 95% CI 2.449–3.864) and in those with malignancy (HR 1.814; 95% CI 1.562–2.107). Notably, a significant interaction was observed with neutropenia status on day 0 or 1 (HR 2.408; 95% CI 2.060–2.815 for non-neutropenia subgroup, and HR 1.346; 95% CI 1.061–1.707 for neutropenic subgroup; P < 0.001). Additionally, when stratified by ARF etiology, shock remained significantly associated with increased hospital mortality in pulmonary (HR 2.912; 95% CI 2.282–3.715), cardiogenic (HR 2.737; 95% CI 2.060–3.635), and sepsis-related ARF (HR 1.377; 95% CI 1.089–1.740), with a significant interaction between ARF etiology and shock (P for interaction <0.001; Fig 2).

## Discussion

In this study, we evaluated the prognostic impact of shock at ICU admission in overall patients with ARF. We found that shock was present in nearly one-fifth of patients (18.6%) and was independently associated with increased ICU mortality (aHR 1.84) and hospital mortality (aHR 1.58) after adjusting for illness severity and comorbidities. This association was consistent across subgroups stratified by SOFA score, respiratory support modality, and neutropenia status. Additionally, a significant interaction with neutropenia status and shock was found, with the relative effect of shock being greater in non-neutropenic patients.

Shock is an acute circulatory failure characterized by impaired tissue perfusion and reduced cellular oxygen delivery, leading to organ dysfunction and elevated lactate levels, with reported short-term mortality rates ranging from 20% to 50% [15–17]. Previous studies have predominantly investigated the association between shock and mortality in selected populations, particularly patients with sepsis or ARDS. Recent meta-analysis informing the Sepsis-3 definitions reported hospital mortality exceeding 40% among patients with septic shock, reflecting the combined effects of circulatory failure and metabolic derangements [18]. Additionally, in the LUNG SAFE study, hospital mortality increased from 34.9% in mild ARDS to 46.1% in severe ARDS, paralleling a significant increase in non-pulmonary SOFA scores highlighting the association between extra-pulmonary organ dysfunction and adverse outcomes in ARDS [19]. Extending these observations, our study demonstrates that the adverse prognostic significance of shock is not confined to sepsis or ARDS-specific populations but persists across a broad and heterogeneous cohort of patients with acute respiratory failure, including those managed with noninvasive respiratory support.

Shock reflects systemic hypoperfusion that may dominantly affect prognosis in acute respiratory failure beyond the severity alone [7,18,20]. In our cohort, patients with shock had substantially higher lactate levels (4.9 vs 1.9 mmol/L) and markers of organ dysfunction, including elevated creatinine, bilirubin, and INR, indicating that circulatory failure was accompanied by widespread metabolic derangement. This likely explains why shock predicted mortality regardless of initial respiratory support modality. Indeed, 63.9% of shock patients on invasive mechanical ventilation had similarly poor outcomes to those managed with HFNC, suggesting that the hemodynamic insult itself, rather than respiratory failure severity, drove adverse outcomes.

Shock-associated inflammation likely contributes to the poor outcomes we observed. In our cohort, patients with shock had markedly elevated CRP (12.4 vs. 6.4 mg/dL) and procalcitonin (7.5 vs. 0.6 ng/mL), suggesting substantial systemic inflammation [21,22]. Prior studies have linked elevated inflammatory markers to endothelial dysfunction and remote organ injury in critically ill patients, which may explain the transition from respiratory failure to multi-organ dysfunction in shock patients [23].

Furthermore, shock patients had a substantially more positive cumulative fluid balance during the early ICU period. Although early fluid resuscitation is essential for restoring intravascular volume and tissue perfusion, accumulating evidence indicates that positive fluid balance is independently associated with worse outcomes in critically ill patients, including prolonged mechanical ventilation, pulmonary edema, and increased mortality [24,25]. The interplay between systemic inflammation, tissue hypoperfusion, and volume overload may collectively contribute to the poor outcomes observed in ARF patients with shock.

Our subgroup analyses revealed consistent associations between shock and mortality across illness severity and respiratory support modalities, and a significant interaction was observed between shock and neutropenia status. The relative prognostic impact of shock, expressed as the hazard ratio, was greater in non-neutropenic patients than in neutropenic patients. These results may reflect differences in baseline risk. Neutropenic patients had a high mortality even in the absence of shock, which may attenuate the relative contribution of shock in this subgroup, whereas in non-neutropenic patients, who had a lower baseline mortality, the addition of shock was associated with a proportionally larger increase in risk [26,27]. Importantly, absolute mortality remained highest among neutropenic patients with shock [28]. As our study was not designed to elucidate the mechanisms underlying this interaction, this finding should be regarded as hypothesis generating and warrants confirmation in dedicated studies. In addition, the high prevalence of malignancy in our cohort merits consideration. This reflects the referral pattern of our institution, a tertiary cancer center, and may influence the generalizability of our findings. Cancer patients may be particularly susceptible to shock-related mortality due to treatment-related immunosuppression, tumor burden, and pre-existing organ dysfunction from chemotherapy [28,29]. In a subgroup analysis stratified by malignancy status, shock remained significantly associated with hospital mortality regardless of malignancy status. The larger relative effect of shock in non-malignancy patients likely reflects their lower baseline mortality, consistent with the ceiling effect observed in the neutropenia subgroup. Together, these findings suggest that the prognostic impact of shock extends beyond cancer-related factors.

The presence of shock at ICU admission has practical implications for ARF management. In our cohort, shock patients received more intensive early treatment; 82.7% received corticosteroids (vs 52.3%), 22.7% required CRRT (vs 8.3%), and norepinephrine and vasopressin were used in over 90% of shock patients. These management differences reflect the clinical reality that shock necessitates immediate attention to circulatory support alongside respiratory management. Our findings support early identification of shock as a trigger for care escalation. Clinicians admitting ARF patients to the ICU may use the presence of vasopressor requirement as a simple marker to identify those at higher risk, guiding decisions about monitoring intensity, organ support planning, and goals-of-care discussions, particularly given the 56.4% hospital mortality rate we observed in this group.

Although this study provides additional information on the association of shock at ICU admission with mortality in patients with ARF, several limitations should be acknowledged. First, this was a retrospective, single-center, nonrandomized cohort study, and thus, residual confounding and selection bias cannot be excluded, limiting causal inference despite multivariable adjustment. Second, shock was defined based on the use of vasoactive agents at ICU admission, which may not capture the full spectrum of circulatory dysfunction, including patients with transient hypotension or occult hypoperfusion not requiring vasopressors. Additionally, we were unable to perform outcome analyses according to specific shock subtypes due to limitations in available data. Consequently, we could not characterize baseline differences or outcomes according to shock subtype, which may have differing pathophysiology and prognostic implications. Third, detailed clinical data regarding the type or severity of shock and acute respiratory failure were insufficient; specifically, information on the timing, dose, and dynamic response to vasoactive therapy was limited, precluding assessment of potential differential effects across varying degrees of circulatory or respiratory compromise. Fourth, our study period encompassed the COVID-19 pandemic, which may have influenced ICU case-mix and respiratory support practices; however, a sensitivity analysis restricted to the non-pandemic period yielded consistent results. Fifth, our cohort included a substantial proportion of patients with hematologic and oncologic malignancies, which may limit the generalizability of these findings to other ICU populations with different case-mix characteristics. Sixth, diabetes was defined based on documented medical history, which may have contributed to the relatively high prevalence observed in our cohort given the characteristics of our tertiary referral center population. However, a sensitivity analysis excluding diabetes mellitus from the multivariable model yielded identical results, suggesting that this did not affect the validity of our findings. Finally, the subgroup analyses were exploratory. Effect modification by SOFA score category, day-0 respiratory support modality, and neutropenia status was evaluated using tests for interaction, but these analyses were not corrected for multiple comparisons. The subgroup findings should therefore be interpreted as hypothesis-generating, and the observed interaction with neutropenia status warrants confirmation in dedicated future studies.

## Conclusion

In conclusion, shock at ICU admission was independently associated with increased ICU and hospital mortality in patients with ARF. The consistent association across clinically relevant subgroups highlights the prognostic importance of early circulatory failure beyond respiratory severity alone. The presence of shock at ICU admission may serve as a readily identifiable marker for risk stratification in this population.

## Supporting information

S1 File STROBE checklist for cohort studies. (DOCX)

S2 Table Comparison of the association between shock and hospital mortality, with and without inclusion of patients admitted during the COVID-19 pandemic period. (DOCX)

S3 Table Univariable and multivariable Cox proportional hazards model for hospital mortality. (DOCX)

S4 Table Univariable and multivariable Cox proportional hazards model for ICU mortality. (DOCX)
