# Supplementary material for: Prognostic impact of shock at ICU admission in acute respiratory failure
Source: PLoS One. 2026 Jul 17;21(7):e0353556. doi: 10.1371/journal.pone.0353556 (PMC13379026; doi:10.1371/journal.pone.0353556)
Supplement: S1 File — (DOCX) [file pone.0353556.s001.docx]

STROBE checklist for cohort studies

*Manuscript: Prognostic impact of shock at ICU admission in acute respiratory failure*

*Manuscript Number: PONE-D-26-13447*

| **Section/Topic** | **Item No.** | **Recommendation** | **Location in manuscript** |
| --- | --- | --- | --- |
| **Title and abstract** | | | |
| **Title and abstract** | 1 | (a) Indicate the study’s design with a commonly used term in the title or the abstract | Line 1; Lines 19–38 |
|  |  | (b) Provide in the abstract an informative and balanced summary of what was done and what was found | Lines 19–38 |
| **Introduction** | | | |
| **Background/rationale** | 2 | Explain the scientific background and rationale for the investigation being reported | Lines 40–55 |
| **Objectives** | 3 | State specific objectives, including any prespecified hypotheses | Lines 56–61 |
| **Methods** | | | |
| **Study design** | 4 | Present key elements of study design early in the paper | Lines 66–68 |
| **Setting** | 5 | Describe the setting, locations, and relevant dates, including periods of recruitment, exposure, follow-up, and data collection | Lines 66–78 |
| **Participants** | 6 | (a) Give the eligibility criteria, and the sources and methods of selection of participants. Describe methods of follow-up | Lines 69–78 |
|  |  | (b) For matched studies, give matching criteria and number of exposed and unexposed | Not applicable |
| **Variables** | 7 | Clearly define all outcomes, exposures, predictors, potential confounders, and effect modifiers. Give diagnostic criteria, if applicable | Lines 92–103; Lines 106–108; Lines 122–126 |
| **Data sources/ measurement** | 8* | For each variable of interest, give sources of data and details of methods of assessment (measurement). Describe comparability of assessment methods if there is more than one group | Lines 89–103 |
| **Bias** | 9 | Describe any efforts to address potential sources of bias | Lines 122–126 |
| **Study size** | 10 | Explain how the study size was arrived at | Lines 66–70 |
| **Quantitative variables** | 11 | Explain how quantitative variables were handled in the analyses. If applicable, describe which groupings were chosen and why | Lines 27–31 |
| **Statistical methods** | 12 | (a) Describe all statistical methods, including those used to control for confounding | Lines 122–126 |
|  |  | (b) Describe any methods used to examine subgroups and interactions | Lines 127–129 |
|  |  | (c) Explain how missing data were addressed | Lines 114–116 |
|  |  | (d) If applicable, explain how loss to follow-up was addressed | Lines 106–108 |
|  |  | (e) Describe any sensitivity analyses | Lines 133–135 |
| **Results** | | | |
| **Participants** | 13* | (a) Report numbers of individuals at each stage of study—eg numbers potentially eligible, examined for eligibility, confirmed eligible, included in the study, completing follow-up, and analysed | Lines 146–147 |
|  |  | (b) Give reasons for non-participation at each stage | Lines 76–78 |
|  |  | (c) Consider use of a flow diagram | Not included |
| **Descriptive data** | 14* | (a) Give characteristics of study participants (eg demographic, clinical, social) and information on exposures and potential confounders | Table 1 (p.8–9) |
|  |  | (b) Indicate number of participants with missing data for each variable of interest | Lines 114–116 |
|  |  | (c) Summarise follow-up time (eg, average and total amount) | Lines 106–108 |
| **Outcome data** | 15* | Report numbers of outcome events or summary measures over time | Lines 209–212 |
| **Main results** | 16 | (a) Give unadjusted estimates and, if applicable, confounder-adjusted estimates and their precision (eg, 95% confidence interval). Make clear which confounders were adjusted for and why they were included | Table 3 (p.12); Lines 122–125 |
|  |  | (b) Report category boundaries when continuous variables were categorized | Lines 97–99; Fig 2 |
|  |  | (c) If relevant, consider translating estimates of relative risk into absolute risk for a meaningful time period | Table 3 |
| **Other analyses** | 17 | Report other analyses done—eg analyses of subgroups and interactions, and sensitivity analyses | Lines 218–221; Lines 232–247; S3 Table |
| **Discussion** | | | |
| **Key results** | 18 | Summarise key results with reference to study objectives | Lines 257–263 |
| **Limitations** | 19 | Discuss limitations of the study, taking into account sources of potential bias or imprecision. Discuss both direction and magnitude of any potential bias | Lines 325–350 |
| **Interpretation** | 20 | Give a cautious overall interpretation of results considering objectives, limitations, multiplicity of analyses, results from similar studies, and other relevant evidence | Lines 273–350 |
| **Generalisability** | 21 | Discuss the generalisability (external validity) of the study results | Lines 340–342 |
| **Other information** | | | |
| **Funding** | 22 | Give the source of funding and the role of the funders for the present study and, if applicable, for the original study on which the present article is based | Funding statement (online submission system) |

*Give information separately for exposed and unexposed groups.

Note: An Explanation and Elaboration article discusses each checklist item and gives methodological background and published examples of transparent reporting. The STROBE checklist is best used in conjunction with this article (freely available on the websites of PLoS Medicine at http://www.plosmedicine.org/, Annals of Internal Medicine at http://www.annals.org/, and Epidemiology at http://www.epidem.com/). Information on the STROBE Initiative is available at www.strobe-statement.org.
