# Supplementary material for: Prognostic impact of shock at ICU admission in acute respiratory failure
Source: PLoS One. 2026 Jul 17;21(7):e0353556. doi: 10.1371/journal.pone.0353556 (PMC13379026; doi:10.1371/journal.pone.0353556)
Supplement: S2 Table — (DOCX) [file pone.0353556.s002.docx]

**S2 Table. Comparison of the association between shock and hospital mortality, with and without inclusion of patients admitted during the COVID-19 pandemic period**

| Variable | N | Univariable analysis HR (95% CI) | Multivariable analysis aHR (95% CI) |
| --- | --- | --- | --- |
| Total period | 3496 | 2.26 (2.00–2.56) | 1.58 (1.37–1.83) |
| Non-pandemic period | 963 | 2.47 (1.96–3.11) | 1.58 (1.23–2.05) |

Values are presented as number (%) or median (interquartile range).

*P* values derived from a multivariable Cox proportional hazards model adjusted for age, reason for acute respiratory failure, Sequential Organ Failure Score, hematologic malignancy, oncologic malignancy, hypertension, diabetes, stroke, chronic kidney disease, heart failure, and chronic obstructive pulmonary disease. The study enrollment period was October 25, 2018 through June 29, 2023 and the COVID-19 pandemic period was defined as February 2020 through May 2023.

Abbreviations: HR, hazard ratio; ICU, intensive care unit
