# Supplementary material for: Prognostic impact of shock at ICU admission in acute respiratory failure
Source: PLoS One. 2026 Jul 17;21(7):e0353556. doi: 10.1371/journal.pone.0353556 (PMC13379026; doi:10.1371/journal.pone.0353556)
Supplement: S4 Table — (DOCX) [file pone.0353556.s004.docx]

**S4 Table. Univariable and multivariable Cox proportional hazards model for ICU mortality.**

| Variable | Univariable HR (95% CI) | *P* | Multivariable HR (95% CI) | *P* |
| --- | --- | --- | --- | --- |
| Shock | 2.86 (2.47−3.31) | <0.001 | 1.84 (1.56−2.17) | <0.001 |
| Age | 1.01 (1.00−1.01) | 0.029 | 1.01 (1.00−1.01) | 0.005 |
| Reason for acute respiratory failure |  |  |  |  |
| Pulmonary | 0.85 (0.70−1.04) | 0.121 | 0.78 (0.63−0.96) | 0.021 |
| Sepsis-related | 1.4 (1.15−1.72) | < 0.001 | 0.64 (0.51−0.81) | < 0.001 |
| Post-operative | 0.28 (0.12−0.63) | 0.002 | 0.39 (0.17−0.89) | 0.026 |
| Others* | 0.68 (0.54−0.85) | < 0.001 | 0.76 (0.6−0.97) | 0.026 |
| SOFA score | 1.20 (1.18−1.22) | < 0.001 | 1.19 (1.17−1.21) | < 0.001 |
| Comorbidities | 1.51 (1.17−1.94) | 0.001 | 1.25 (0.93−1.66) | 0.135 |
| Hematologic malignancy | 1.34 (1.11−1.61) | 0.002 | 1.01 (0.83−1.23) | 0.913 |
| Oncologic malignancy | 2.05 (1.75−2.4) | < 0.001 | 1.98 (1.65−2.37) | < 0.001 |
| Hypertension | 0.82 (0.71−0.96) | 0.011 | 0.71 (0.60−0.86) | < 0.001 |
| Diabetes | 1.49 (1.16−1.93) | 0.002 | 1.26 (0.94−1.68) | 0.119 |
| Stroke | 0.74 (0.58−0.95) | 0.017 | 0.86 (0.66−1.1) | 0.231 |
| Chronic kidney disease | 1.08 (0.87−1.33) | 0.492 | 0.94 (0.75−1.19) | 0.614 |
| Heart failure | 0.95 (0.77−1.18) | 0.663 | 1.13 (0.90−1.43) | 0.285 |
| COPD | 1.0 (0.77−1.30) | 0.984 | 1.2 (0.91−1.57) | 0.191 |

Values are presented as number (%) or median (interquartile range).

*P* values derived from a multivariable Cox proportional hazards model adjusted for age, reason for acute respiratory failure, Sequential Organ Failure Score, hematologic malignancy, oncologic malignancy, hypertension, diabetes, stroke, chronic kidney disease, heart failure, and chronic obstructive pulmonary disease.

*Others include neuromuscular disease, multiple trauma and unclassified causes.

Abbreviations: CI, confidence interval; COPD, chronic obstructive pulmonary disease; HR, hazard ratio; ICU, intensive care unit
